# Inflammation in the Tumor Microenvironment

**DOI:** 10.1155/2018/1965847

**Published:** 2018-06-24

**Authors:** Qing Lin, Shi Jin, Mei Han, Wenxin Zheng, Jiaming Liu, Xiaolong Wei

**Affiliations:** ^1^Department of Anesthesiology and Critical Care Medicine, Johns Hopkins University School of Medicine, Baltimore, MD 21205, USA; ^2^Division of Gastroenterology, Department of Pediatrics, University of Florida, Gainesville, FL 32610, USA; ^3^McKusick-Nathans Institute of Genetic Medicine, Johns Hopkins University School of Medicine, Baltimore, MD 21205, USA; ^4^Ludwig Center for Metastasis Research, Department of Radiation and Cellular Oncology, The University of Chicago, Chicago, IL 60637, USA; ^5^Department of Orthopedic Surgery, The First Affiliated Hospital of Nanchang University, Nanchang, Jiangxi 330006, China; ^6^Shantou University Medical College, Shantou, Guangdong 515041, China

The objective of this special issue is to provide a better understanding of the tumor-associated inflammatory signaling and their microenvironmental cross talk and network, which is crucial for elucidating tumorigenesis mechanisms and for cancer therapy, prevention, and risk assessment. A substantial number of manuscripts were submitted, and after a thorough peer review process, eleven papers were accepted for inclusion in this special issue. These papers investigate the involvements of the essential microenvironmental elements including inflammatory mediators and cellular effectors in the context of cancer-related inflammatory processes; they also offer new insights into tumor immunotherapy, which will help explore new targets or approaches for cancer treatment, by either boosting antitumor immunity or disrupting tumor-educated immunosuppression.

The paper by S. Ye et al. investigates the immunoregulatory properties of enavatuzumab, a humanized anti-TWEAK receptor monoclonal antibody. This preclinical study demonstrates that enavatuzumab exerted its potent antitumor activity by actively recruiting and activating myeloid effectors to kill tumor cells. The findings of enavatuzumab-induced chemokines warrant further evaluation in clinical studies as potential biomarkers for such activity.

The paper by M. Janiczek et al. presents a systematic review on immunotherapy as a promising treatment for prostate cancer. The authors summarize the recent advances and trends on three categories of immunotherapies: checkpoint inhibitors, cytokines, and therapeutic cancer vaccines, providing better understanding on the complexity of tumor cells and their interactions with the surrounding inflammatory microenvironment.

The paper by W. Zeng et al. tries to dissect the mechanism underlying the effects of methylation agent decitabine (DAC) treatment on myeloid myelodysplastic syndromes (MDS) by in vitro study with the MDS cell line SKM-1. Their results show that DAC could rescue FOXO3A, a potentially tumor-suppressive transcription factor. The reactivation of FOXO3 signaling is also critical for the anti-MDS therapeutic effects of DAC.

The paper by M. Liu et al. conducts bioinformatics analysis on differentially expressed genes (DEGs) between tumor samples and normal samples to identify key genes and pathways in glioma. Some identified hub genes may be important for regulating inflammatory responses, and CDK17, GNA13, PHF21A, and MTHFD2 might be important and potentially valuable in the prognosis and treatment of glioma. These identified genes and pathways would provide a more detailed molecular mechanism for underlying glioma initiation and development.

The paper by G.-Y. Liou focuses on cytokine signaling of tumor-facilitated immune cells and of cancer cells that lead to tumor initiation, progression, and metastasis of pancreatic cancer and prostate cancer. The review of the complex cytokine network among tumor cells, immune cells, and other types of cells including stromal cells and endothelial cells would provide information on developing effective immunotherapies for treating related tumors.

The paper by G. D'Arena et al. summarizes published data on the prognostic significance of regulatory T cells in hematological malignancies. The authors attribute the variability reported by different groups to the heterogeneity of the experimental approaches, and emphasize the need to apply standardized approaches in the study of regulatory T cells in hematological malignancies and in cancer in general.

S. Chen et al. conducted a large cohort study to validate the involvement of four genes, STAT1, IGF1, RAC1, and MDM2, in the recurrence of a giant-cell tumor of the bone. Their findings suggest the potential of these genes to serve as biomarkers for the recurrence of the giant-cell tumor of the bone. Moreover, data presented also indicate that immune response mediated by these four genes through their interacting proteins might play important roles in the recurrence of the giant-cell tumor of the bone.

The paper by A. Aponte-López et al. discusses the evidence supporting protumoral and antitumoral roles of mast cells in breast cancer progression, highlighting recent findings placing mast cells as important drivers of tumor progression, as well as the potential use of these cells or their mediators as therapeutic targets. The paper calls for more work to clarify the role of mast cells in breast cancer and for a better understanding of mast cell communication with tumor cells and other immune cells within the tumor stroma.

The paper by G. Chen et al. compared the clinicopathologic features of gastric cardia cancer (GCC) and esophageal cancer (EC). Results showed that GCC in the Chaoshan high-risk area in China displays clinicopathologic characteristics different from those of EC, although they share genetic risk factors and similar geographic aggregation. Studies also detected toll-like receptor- (TLR-) 4 expression in gastric cardia epithelial cells and demonstrate upregulated TLR4 expression in gastric cardia inflammation and GCC, suggesting that TLR4 plays a role in GCC carcinogenesis.

The paper by Y. Xie et al. briefly reviews the mechanism underlying immunosuppression in hepatocellular carcinoma (HCC), followed by a summary of major immunotherapeutic approaches, including adoptive cell therapy, tumor vaccines, immune checkpoint inhibitors, and oncolytic virotherapy, for HCC with their current advances. The pros and cons of each option for HCC treatment are also discussed in this review article.

The paper by Q. Xu introduces a novel inflammation-based prognostic score, the fibrinogen/albumin ratio, which could predict prognoses of HCC patients undergoing curative resection. With a retrospective study, their data demonstrate that an elevated fibrinogen/albumin ratio significantly correlates with poorer survival and a higher risk of recurrence in HCC patients. The potential of the fibrinogen/albumin ratio to be a promising serum biomarker for predicting HCC prognoses is indicated in this study.

All the topics highlighted above, we believe, would be of particular interest to the readers, especially the basic and clinician scientists who specialize in immunology and immunooncology. Inflammation in the tumor microenvironment affects every aspect of tumor development, including initiation, promotion, malignant conversion, invasion, and metastasis, as well as response to therapy. In turn, to elucidate the signaling network of an inflammatory tumor microenvironment would be also crucial for developing a novel effective therapy for some complex inflammatory diseases, such as the pulmonary arterial hypertension (PAH) which is a fatal and multifactorial disease caused by vascular inflammation. Given the striking pathogenic analogies between cancer and PAH, as pulmonary vascular cells acquired cancer traits including apoptosis-resistant excessive proliferation during PAH development, the cancer theory of PAH is recently developed [1], and application of antineoplastic drugs thus may be a promising way to tackle established PAH [1]. Nevertheless, there is still a long way to go before a clear picture of the inflammation-tumor cross talk can be accomplished, and this special issue is adding a few new points in the picture being painted.
